# Finite element modeling to predict the influence of anatomic variation and implant placement on performance of biological intervertebral disc implants

**DOI:** 10.1002/jsp2.1307

**Published:** 2023-12-27

**Authors:** Maho Koga, Byumsu Kim, Marianne Lintz, Sertaç Kirnaz, Jacob L. Goldberg, Ibrahim Hussain, Branden Medary, Kathleen N. Meyers, Suzanne A. Maher, Roger Härtl, Lawrence J. Bonassar

**Affiliations:** ^1^ Meinig School of Biomedical Engineering Cornell University Ithaca New York USA; ^2^ Sibley School of Mechanical and Aerospace Engineering Cornell University Ithaca New York USA; ^3^ Weill Cornell Medicine New York New York USA; ^4^ Hospital for Special Surgery New York New York USA

**Keywords:** cervical spine, finite element analysis, intervertebral disc implant, large animal model

## Abstract

**Background:**

Tissue‐engineered intervertebral disc (TE‐IVD) constructs are an attractive therapy for treating degenerative disc disease and have previously been investigated in vivo in both large and small animal models. The mechanical environment of the spine is notably challenging, in part due to its complex anatomy, and implants may require additional mechanical support to avoid failure in the early stages of implantation. As such, the design of suitable support implants requires rigorous validation.

**Methods:**

We created a FE model to simulate the behavior of the IVD cages under compression specific to the anatomy of the porcine cervical spine, validated the FE model using an animal model, and predicted the effects of implant location and vertebral angle of the motion segment on implant behavior. Specifically, we tested anatomical positioning of the superior vertebra and placement of the implant. We analyzed corresponding stress and strain distributions.

**Results:**

Results demonstrated that the anatomical geometry of the porcine cervical spine led to concentrated stress and strain on the posterior side of the cage. This stress concentration was associated with the location of failure of the cages reported in vivo, despite superior mechanical properties of the implant. Furthermore, placement of the cage was found to have profound effects on migration, while the angle of the superior vertebra affected stress concentration of the cage.

**Conclusions:**

This model can be utilized both to inform surgical procedures and provide insight on future cage designs and can be adopted to models without the use of in vivo animal models.

## Graphical abstract

A finite element model of the cervical porcine spine was created to simulate loading of intervertebral disc implants. In addition, we analyzed the effect of placement of the cage and joint angle on stress and strain distribution as well as migration of the implant.

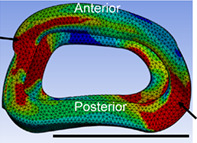

## 1 INTRODUCTION

Recent successes of cellular and tissue engineering (TE) have provided avenues for potential treatment of degenerative disc disease and have investigated TE applications targeting both the annulus fibrosis (AF) and nucleus pulposus (NP) of the intervertebral disc (IVD).^1^, ^2^, ^3^, ^4^, ^5^, ^6^, ^7^, ^8^, ^9^ A composite cell‐biomaterial IVD implant was created utilizing collagen as a contractile scaffold for the AF, with a cross‐linked alginate gel for the NP.^2^, ^10^, ^11^, ^12^, ^13^ The efficacy of these implants has been proven in vivo in small and large animal models; previous in vivo studies in rat tails,^10^, ^12^, ^14^, ^15^, ^16^ lumbar canine models,^2^ and caprine lumbar model^17^ demonstrated an ability to maintain disc height and hydration after implants reach maturity. However, despite such advances, TE‐IVDs need initial stabilization for maturation. Previous studies have found that a fraction of samples was unstable upon implantation, therefore utilized stainless steel plates to stabilize the implants.^9^, ^18^ In addition, an ex vivo cervical model demonstrated added stability from the plate.^19^ Collectively, this data suggest TE‐IVD structures require additional mechanical support. One such avenue for stabilizing the implants is to utilize a rigid cage construct to support the TE‐IVDs until they reach maturation.^20^

Common methodology for restoring disc height includes interbody spinal fusion and implants for total disc replacements, utilizing various metals, polymers, and bone grafts.^21^ However, current procedures present risks that may require subsequent operations to address or to explant hardware.^22^, ^23^ In contrast, bioresorbable and biodegradable materials may mitigate the need for secondary surgery due to complications, since they do not corrode and are less stiff.^21^, ^23^, ^24^ Biodegradable and bioresorbable polymer‐based materials have illustrated utility in spinal surgery, demonstrating several advantages over traditional metal implants.^21^, ^23^, ^24^, ^25^ One of the most promising materials for this application is polylactide acids (PLA), as it is biocompatible and provides enough initial support necessary for spinal implants.^1^, ^23^, ^26^

While in vivo experimentation is valuable in providing insight on implant behavior and performance, surgical testing is often expensive and time‐consuming. Computational simulations utilizing finite element (FE) analysis can aid in our understanding of material properties and systems of high complex geometry,^27^, ^28^, ^29^ and provide biomechanical insight into the feasibility of materials as implants.^28^, ^30^ FE analysis is particularly attractive in this case to test a range of parameters that may be critical to surgically implanted TE‐IVDs.^31^, ^32^ Previous attempts at modeling cage structures with simplified geometries have been performed to analyze stress distribution and displacement, but none have focused on predicting the mechanical performance and failure of cage structures in the cervical spine.^27^, ^31^, ^32^, ^33^, ^34^, ^35^, ^36^, ^37^ Furthermore, to the best of our knowledge, there has not been any simulations which utilized the unique geometry of the porcine cervical spine to understand the mechanical behavior of the cage in vivo. Therefore, capturing the anatomical geometry and simulating in vivo loading achieves a model that closely evaluates cage stability under non‐uniform loading. Such a model can be instrumental in future cage designs^38^ and proves imperative for informing surgeons on the placement of TE‐IVD constructs for restoring intervertebral height.

As such, the objectives of this study were to (1) create and test in vivo cage design to stabilize TE‐IVD implants in the porcine spine, (2) create a FE model to understand and simulate the behavior of the IVD cages under compression in vivo, (3) validate the FE model specific to the anatomy of the porcine cervical spine, and (4) demonstrate the usability of the validated model to predict the effects of implant location and vertebral angle of the motion segment on implant behavior.

## 2 MATERIALS AND METHODS

### 2.1 Design of cages

PLA cages were designed and produced in Autodesk Inventor Professional® 2021 (Autodesk Inc., San Rafael, CA). Cages were printed from Prusament PLA using an Original Prusa i3 MK3S+ (Prusa Polymers, Czechia) and a E3D V6 0.25 mm nozzle (E3D, UK) with a layer height of 0.05 mm. The dimensions of the cage were 14 × 9 × 3 mm.

### 2.2 Surgical procedure

**FIGURE 1:**
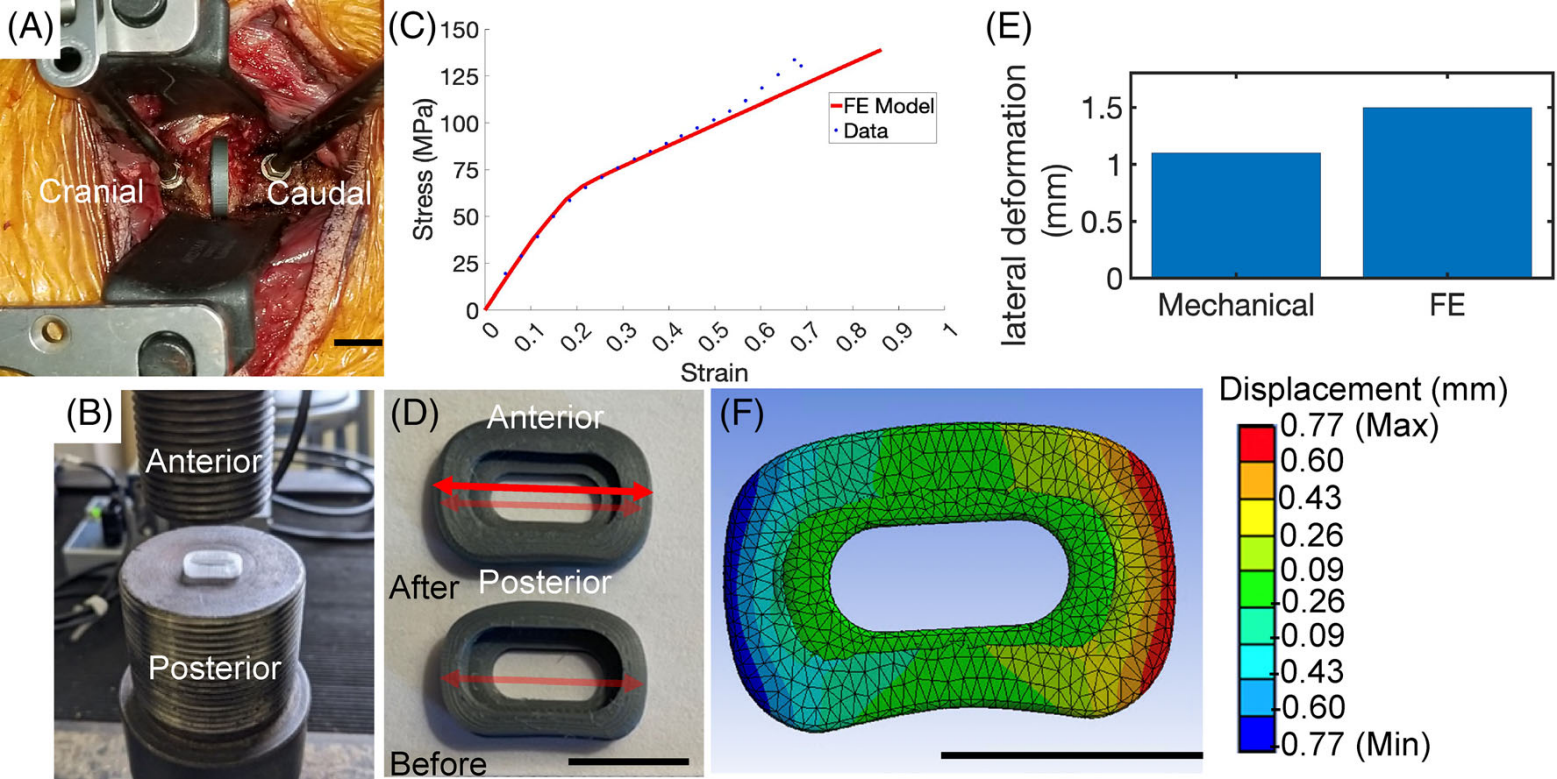
Experimental data for in vitro FE model validation. All scale bars represent 1 cm. (A) Surgical set‐up of cages tested in vivo in 2‐ to 3‐year‐old male Göttingen minipigs. (B) Mechanical testing set‐up of IVD cage. (C) Stress versus strain graph. Red line represents data collected from FE model, and blue dots are from mechanical testing. *R* ^2^ value between the two data sets for the elastic region was 0.998 and was 0.993 for the tangent moduli. Normalized RMSE error was 6.62%. (D) Image of cages before and after compression with scale bar. Faint red arrow shows measurement of cage before compression, and solid red arrow shows measurement after compression. The dimensions of the cage are 14 × 9 × 3 mm before compression, with lateral expansion of 1.1 mm after compression. (E) Bar graph of lateral movement (mm) of mechanical testing data and FE model data. (F) Image of the cage after compression simulation in FE model. Displacement values are only for compression in the lateral direction. *Source*: Image F courtesy of Ansys, Inc.

All procedures were reviewed and approved by the appropriate Institutional Animal Care and Use Committee (IACUC). Discectomy was carried out on two, 2–3 year‐old skeletally mature Göttingen minipigs (Marshall BioResources, North Rose, NY). Cages were implanted in the empty support structure at levels C5–C6 to test the most difficult curvature, with C3–C4 left as the healthy control for 4 weeks. One animal received implants at both C3–C4 and C5–C6 levels (Figure 1A). Animals were anesthetized after appropriate fasting and underwent endotracheal intubation prior to being attached to the anesthesia and ventilator units. General anesthesia was maintained throughout the procedure using anesthetic agents, and the animal's vital signs (heart and respiration rate, oxygenation, body temperature, and invasive blood pressure when needed) were continuously monitored. Upon obtaining proper depth of anesthesia, the ventral neck was prepared for surgery using povidone iodine followed by 70% isopropyl alcohol.

All surgical procedures followed the spinal cord injury (SCI) guidelines for large animal surgery, including sterilization, pre‐ and postoperative care, and surgical procedures were carried out as previously described.^2^ Animals were placed on their backs with their necks hyperextended and secured to the table. A 3–4‐inch ventral midline incision was made from the base of the larynx to the sternum in each minipig, after which the trachea was exposed by separating the paired sternocleidomastoideus and sternohyoideus muscles. Experimental levels were identified by exposing the paired longus colli muscle and were marked by cauterizing of the anterior ligaments. Small, curved hemostats were used to separate the long colli muscle overlying the ventral AF.

The following steps were carried out microscopically. After incisions of the ventral annulus were made with a scalpel, the inner AF and NP were extracted using a small tartar scraper or 3–0 to 4–0 bone curette. The outer posterior portion of the annulus was left intact. Cage constructs were implanted in experimental groups in the disc space and the outer annulus was closed with suture material. Bipolar cauterization was used for homeostasis, and the wounds were closed with absorbable subcutaneous and cutaneous sutures. Incision sites were dressed in accordance with standard veterinary practices and in consultation with an SCI veterinarian. Once gag reflex returned, animals were extubated, and anesthesia was discontinued. Animals received antibiotics peri‐operatively and were recovered by veterinary staff in SCI large animal housing. Postoperative pain management was administered as prescribed by an SCI veterinarian. Orthopedic and neurologic exams were performed by an SCI veterinarian at least once daily for the first three postoperative days, who was available for consultation for additional examinations. X‐ray imaging was utilized to monitor implants preoperatively, immediately postoperatively, and at weekly intervals by assessing disc height and cage position. Assessment of disc height was done by determining the disc height index (DHI), or the ratio of disc height by adjacent vertebral body height, as previously described.^2^ During imaging, animals were anesthetized as described above. Constructs were harvested at 4 weeks.

### 2.3 Mechanical testing of cages

Mechanical data were collected for validation of the FE model and to fit the model (Figure 1B,C). Cages were uniaxially compressed under quasi‐static conditions between flat plates using a Screw‐drive, uniaxial tension/compression tester (United Testing Systems, Model FM‐20), with a triangular wave form at 0.1 mm/s and sampling frequency of 50 Hz. Cages were tested at the load cut‐off of instrument at 2000 pounds (lbs) based on predicted material failure. Cages were not fixed during compression. The vertical displacement of the top plate was measured, and the displacement of the IVD cage as a function of load was calculated by taking the point where load reads below 0 as the first point of contact of the cage with the rod. Deformation in the lateral direction was analyzed, where width of a compressed cage and a non‐compressed cage were measured (Figure 1D). We estimated the following materials parameters of PLA from the results: Young's modulus: 450 MPa, Poisson's Ratio: 0.36, Yield Strength: 65 MPa, and hardening coefficient: 130 MPa.

### 2.4 FE simulations

All simulations were conducted using Ansys® Academic Research Mechanical, 2021R (Ansys Inc., Canonsburg, PA). PLA was modeled as an isotropic elastic and bilinear isotropic hardening material to simulate both elastic and plastic deformation, and material properties estimated from the mechanical testing data were used.

### 2.5 FE model validation

Validation was conducted by fitting the mechanical testing data to our model by setting a −1.8 mm directional displacement on the top surface of the cage with fixed boundary conditions on the bottom surface and measuring deformation in the lateral direction to capture material properties (Figure 1D,E). We chose −1.8 mm because −2 mm displacement caused the mesh structure to start collapsing and overlapping with each other due to the contact of rigid bodies. For contact modeling simulations with vertebra and cage, displacement of the superior vertebra was modeled as displacement controlled rather than load controlled due to error in modeling when setting an applied force. To keep the applied force consistent across iterations, applied displacement was adjusted based on the calculated average force on the top surface of the cage for all analysis. Average applied force on the IVD cage was calculated by averaging the stress on the top surface of the IVD cage and multiplying by the surface area of the top of the cage.

### 2.6 FE model setup with cervical vertebra anatomy

Porcine cervical vertebra motion segments were retrieved from Computed Topography (CT) scans using Avizo® Software (Thermo Fisher Scientific™ Inc., Waltham MA). A segment of the mini pig cervical spine (C3) was isolated from the CT scan and imported into Ansys® SpaceClaim. The vertebra was duplicated to form the superior and inferior vertebrae and truncated for simplicity. The model was composed of duplicated C3 segments rather than C3/C4 segments to keep the model non‐specific to any part of the spine, due to anatomical variation in the cervical spine with location. Our result showed that there was no statistically significant difference in cage stress‐distribution between C3/C3 and C3/C4 segments (Supporting Information 1). Vertebral bone was modeled with a Young's modulus of 17 GPa, an average of trabecular and cortical bone, and Poisson's ratio of 0.3.^39^, ^40^, ^41^, ^42^ We selected a body mesh with tetrahedral elements with element size of 1 mm for the vertebrae, and 0.3 mm for the cage after mesh convergence analysis (Supporting Information 2 and 3). Contact between the superior vertebra and the cage was set as frictionless contact. The inferior vertebra was set as a fixed support, and the two sides of the cage were set as frictionless support to allow for sliding in the anterior–posterior axis. Displacement of the superior vertebra was initially set to 2 mm and adjusted to simulate an average applied compressive force of 2100–2400 N based on physiological loading on a single vertebra, utilizing values for lumbar spine to test higher stress values.^43^ We analyzed the Von‐Mises equivalent stress, strain, and total deformation of the cage under compression.

**FIGURE 2:**
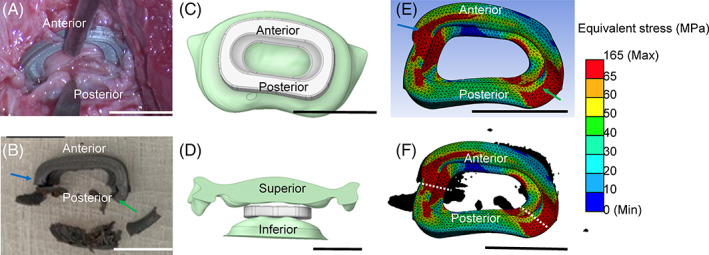
Comparison of in vivo failure to FE prediction of stress distribution. All scale bars represent 1 cm. (A) IVD cage implant in vivo. (B) Image of IVD cage after 4 week in vivo experiment. (C) Simulation top view of inferior vertebra. Set‐up of FE model with IVD cage placed with bottom on inferior vertebra. (D) Simulation view of IVD cage implant from posterior side. (E) FE model results of IVD cage after 2 mm applied displacement, with yield strength of PLA modeled at 65 MPa. (F) FE model results overlaid on in vivo results (2B) and dotted line drawn over where cage failed in vivo. *Source*: Images C–F courtesy of Ansys, Inc.

The FE model was then used to simulate two scenarios of compression for the cages: (1) uniform compression with flat contact to mimic mechanical testing, and (2) with the anatomic geometry of the porcine spine. Under uniaxial compression, stress of the cage was calculated by taking the average stress on the top surface of the cage, and strain (Δ*L*/*L*_0_) was calculated using the displacement (Δ*L*) data from the FE model and using the original height of the cage as *L*_0_. Deformation in the x‐direction was also compared (Figure 1D,E,F). In vivo data were compared against results from FE modeling (Figure 2A,B).

This model was further utilized to predict the mechanical behavior of the implant with different placement and joint angle of the spine. We studied the different placements of the cage with respect to the anterior–posterior axis in 0.2 mm increments from 0 to 0.8 mm, and the angle of the superior vertebra in 2° increments for a total range of 14° of rotation to simulate the in vivo range of the cervical spine.^44^, ^45^ The original angle of the superior vertebra was visually predicted to be 6°. We conducted nodal stress and strain distribution analyses for both different positions of the cage and angles of superior vertebra by comparing the magnitude of displacement to account for displacement in all directions.

## 3 RESULTS

Post‐operative assessment with x‐ray imaging indicated that cages were intact, and no adverse neurological symptoms were present (Supporting Information 4). Cages were intact in the cervical spine and remained intact until they were removed at 4 weeks. However, there was structural damage to the cages in the right posterior side and the left anterior side of the implant upon removal from the porcine spine (Figure 2B, Supporting Information 4). Additionally, there was warping of the cage on the surface, particularly along the lip of the anterior side. Migration of the cages was also observed.

The force‐displacement curves generated from mechanical testing data were used to extract material properties used as inputs for the FE model and used for the validation of the model (Figure 1C). Testing data and FE model matched well up to stress of 80 MPa, above which experimental stress data was slightly higher than FE simulations. The FE model incorporated two moduli for low and high strain behavior, as well as a transition strain that determined the point at which the material changed from low to high strain behavior. *R* ^2^ value between FE and mechanical testing data for the elastic region was 0.998 and 0.993 for the hardening coefficient. Normalized RMSE error between FE model and mechanical testing data was 6.62% and was calculated by dividing RMS error by the average stress of FE model data and mechanical testing data. Visual inspection of lateral expansion from experiments and FE model were also compared. Photographic measurements showed lateral expansion of 1.1 mm, similar to the 1.5 mm expansion predicted from FE model (Figure 1D,E,F).

Implants were placed in the center of the vertebra in FE simulations (Figure 2C,D), and a ~2100 N load was applied for rotational studies, and ~2300 N for placement (Figure 2E). Stress maps revealed that maximum stress exceeded 150 MPa, and stress was concentrated in the corners of the implant. Regions of the implant that experienced stress higher than the yield strength (65 MPa) were colored in red. Notably, these regions corresponded to locations of failure observed in vivo (Figure 2F).

**FIGURE 3:**
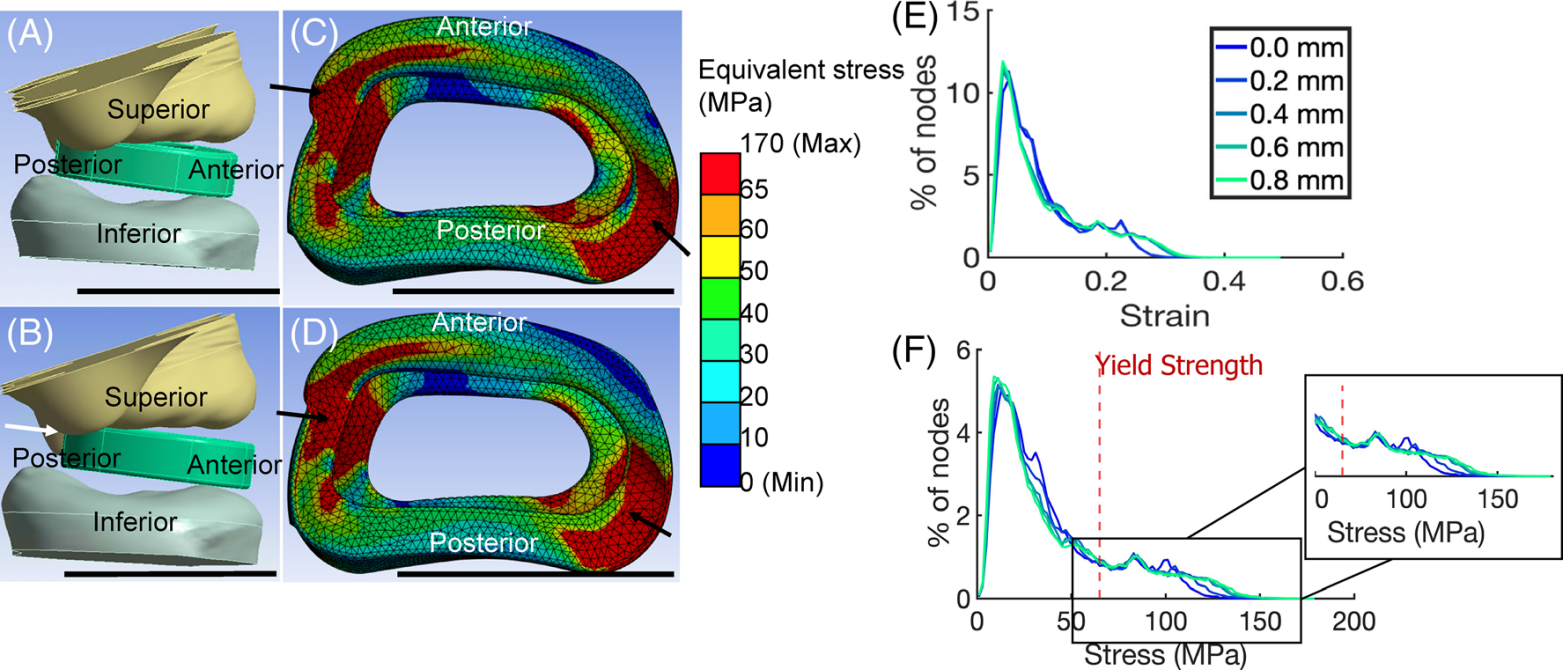
Impact of implant placement on stress and strain distribution. All scale bars represent 1 cm. (A). Set‐up of FE model with 0 mm shift of the IVD cage toward the anterior side. (B). Set‐up of FE with 0.8 mm shift of the IVD cage toward the anterior side. (C). FE model results of equivalent stress (von‐Mises) in MPa of the IVD cage after compression with 0 mm shift in cage. (D). FE model results of equivalent stress (von‐Mises) in MPa of the IVD cage after compression with 0.8 mm shift in cage. (E). Graph of equivalent strain (von‐Mises) with placement of the IVD cage toward the anterior side. (F). Graph of equivalent stress (von‐Mises) with placement of the IVD cage toward the anterior side. *Source*: Images A–D courtesy of Ansys, Inc.

Having validated the FE model and demonstrated that the model predicted regions of implant failure in vivo, we investigated the effects of cage placement on the stress and strain distributions on implants. Simulations were performed on cage locations ranging from the center of the vertebra (0 mm) (Figure 3A) to external alignment with the anterior edge of the vertebral body (0.8 mm) (Figure 3B). Stress distributions showed similar patterns for all locations examined (Figure 3C,D, Supporting Information 5A and B). As cages were placed more anteriorly, the footprint of regions experiencing higher stress and strain expanded slightly. The fraction of nodes experiencing stresses greater than 65 MPa was around 18% for 0.0 mm movement, and steadily increased up to 22% for 0.8 mm movement. (Figure 3E,F).

**FIGURE 4:**
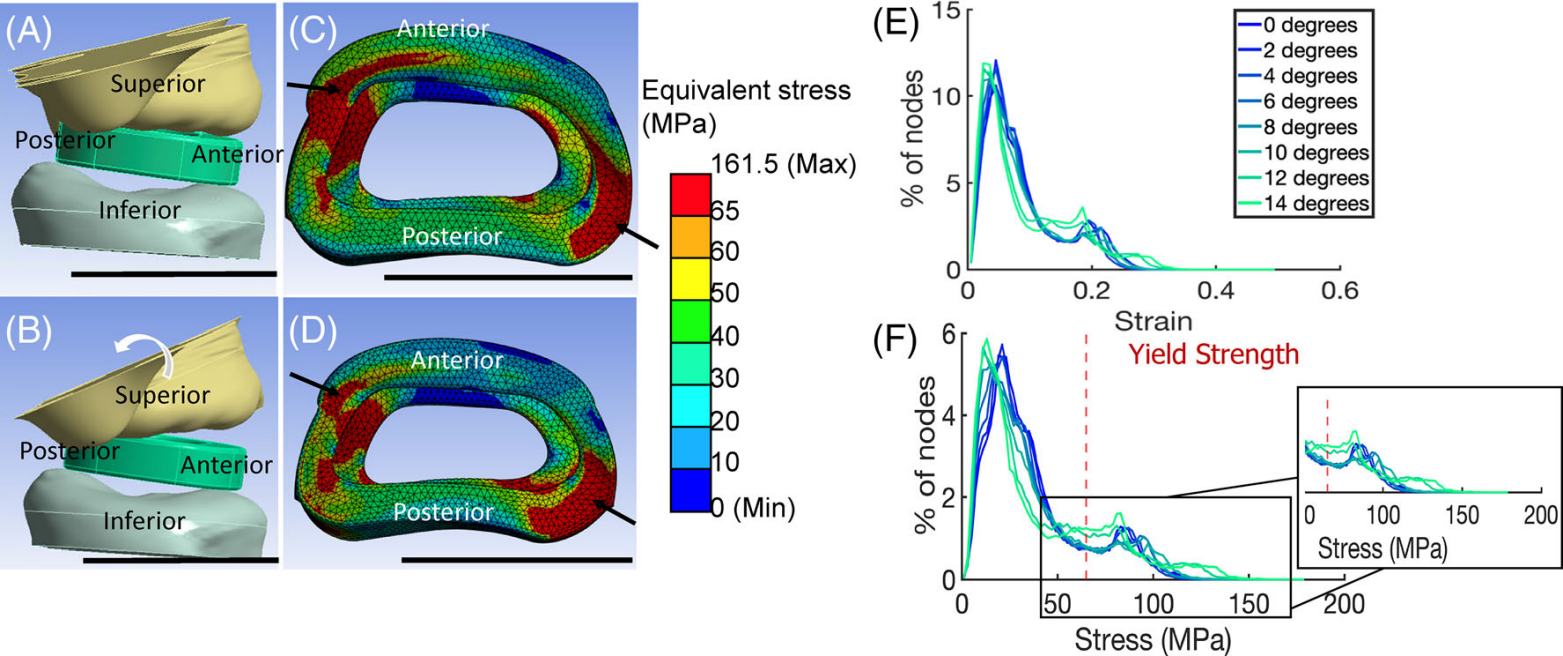
Effect of joint angle on stress and strain distribution. All scale bars represent 1 cm. (A) Set‐up of FE model with 0° rotation of the superior vertebrae toward the posterior side. (B) Set‐up of FE model with 14°rotation of the superior vertebrae toward the posterior side. (C) FE model results of equivalent stress (von‐Mises) in MPa of the IVD cage after compression with 0° rotation. (D) FE model results of equivalent stress (von‐Mises) in MPa of the IVD cage after compression with 14° rotation. (E) Graph of equivalent strain (von‐Mises) with different rotation of superior vertebra. (F). Graph of equivalent stress (von‐Mises) with different rotation of superior vertebra. *Source*: Images A–D courtesy of Ansys, Inc.

We also evaluated the effect of motion segment angle on stress and strain distribution over a range of joint angles from 0° (Figure 4A) to 14° (Figure 4B). Stress distribution was similar across this range of angles, with stress concentrated in the corner of the implants (Figure 4C,D, Supporting Information 5C,D). Increasing joint angle increased stress in the right posterior corner but decreased stress on the left edge. These changes made the distribution of stress more bimodal and at higher rotation angles, stress distributions increased above ~65 MPa. (Figure 4E). The percentage of nodes above 65 MPa (Yield stress) remained around 15%–16% below 8° rotation and increased above 20% for 12 and 14° rotation (Figure 4F).

**FIGURE 5:**
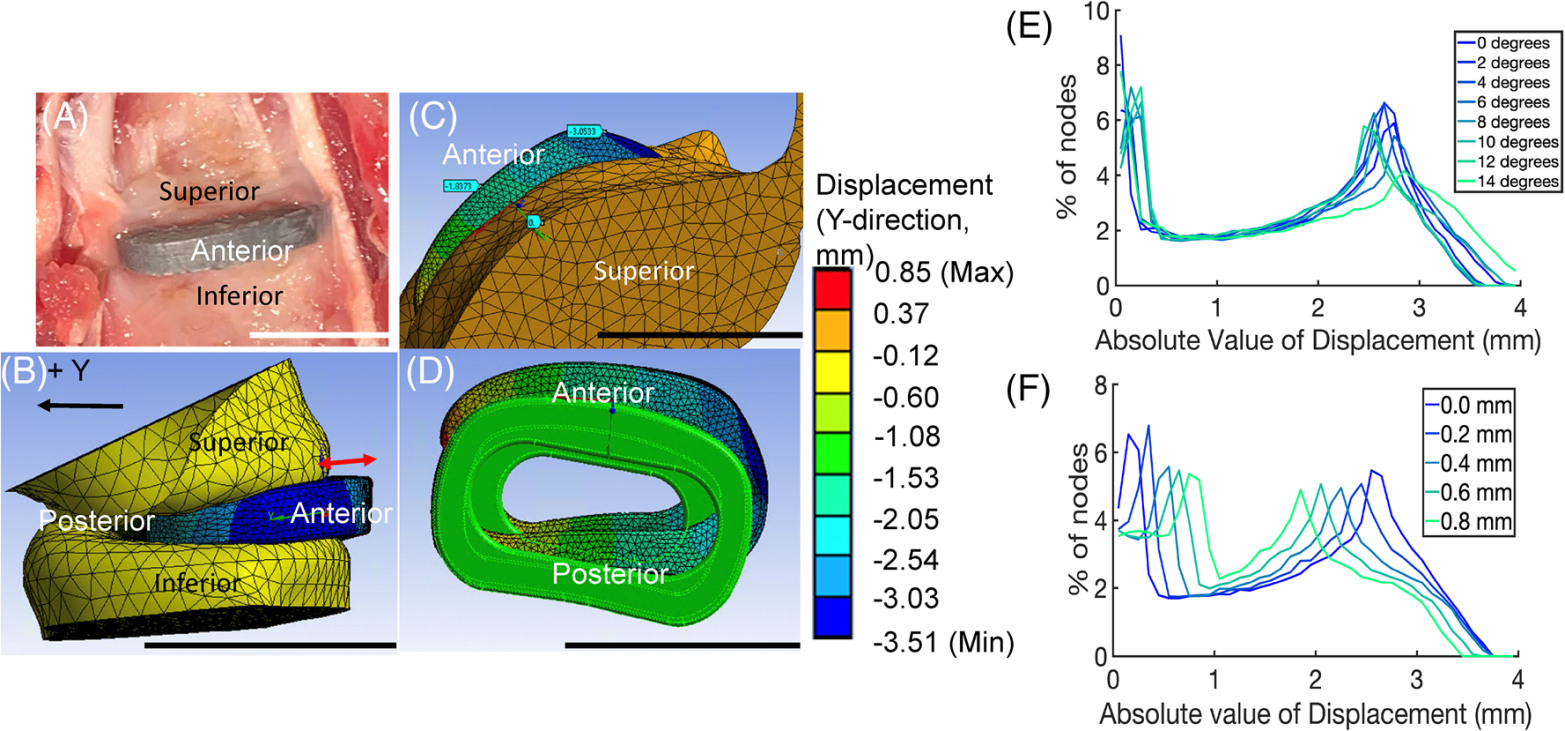
Effect of placement and joint angle on implant migration. All scale bars represent 1 cm. (A). Image of IVD cage implant protruding out of anterior side. (B). Simulation side view of IVD cage implant after 2 mm applied displacement. (C). Simulation superior view of IVD cage implant after 2 mm applied displacement. (D). Before (green) and after visual of cage in FE model after 2 mm displacement. (E). Graph of displacement in the y‐direction only (mm) with different rotation of superior vertebra. (F). Graph of displacement in the y‐direction only (mm) with movement of the IVD cage toward the anterior side. *Source*: Images B–D courtesy of Ansys, Inc.

In vivo results showed anterior migration of implants, often immediately postoperatively (Figure 5A).^2^ The FE model demonstrated both rigid‐body rotation and migration of implants upon loading (Figure 5B,C). The left posterior corner of the cage after compression stayed stationary, however the right anterior side migrated over 4 mm (Figure 5D). This rotation was manifested as bimodal distribution of displacement, with two distinct peaks. The lower peak at 0.1 mm represented the left side of the implant and the higher peak greater than 2 mm described the right side (Figure 5E,F). The location of peak displacements of the left side of the implant moved to higher values as the implant was moved more anteriorly. Furthermore, the right side had lower peak displacement with movement more anteriorly, indicating more uniform movement and less rotation.

## 4 DISCUSSION

We developed a TE‐IVD cage designed to specifically maintain disc height during the initial implantation. When these devices were implanted in the porcine cervical spine, failure occurred in the posterior region. This was surprising given the yield strength of PLA (50–70 MPa)^46^, ^47^, ^48^ greatly exceeds the reported porcine intradiscal pressures (1–2 MPa).^49^ Furthermore, migration of the cage toward the anterior side of the vertebra was observed. A possible explanation of both the failure as well as the migration is the presence of anatomical features of the porcine cervical vertebral endplate^50^, ^51^ that concentrate stress on implants. Thus, to elucidate the failure mode of these implants, a FE model was created that simulated the mechanical behavior of IVD implants in contact with vertebrae. This model utilized the unique geometry of the porcine cervical spine and predicted regions of stress and strain behavior under compression. Regions of high stress concentration predicted by the model mirror the pattern of in vivo failure. We thus utilized this model to understand the effect of joint angle and implant placement of IVD cages on stress distribution and migration of the cage.

From the non‐uniform distribution of stress and strain of the cage seen in vivo, we expected that the geometry of the porcine vertebrae played a significant role in explaining the compressive failure of the cage. Intradiscal pressure of porcine cervical spine,^49^ human lumbar spine,^52^ and other large animals such as sheep^53^ are over 1 MPa. Our FE model predicted that stress on the implants exceeded 150 MPa. As such, material selected for such implants must account for these high stresses. The bony protrusion of the posterior side of the inferior vertebra was a likely source of stress concentration. However, a closer look at the positioning of the bony protrusions illustrated that the localization of the failure to the corners of the cage did not completely align with the location of the protrusions. The simulation demonstrated instead that these bony protrusions did not directly deform the cage, but rather warped the cage and generated torque, leading to cage deformation and rotation. As such, the stress concentration at the corners were likely due to warping of the cage under compression.

Placement of the IVD cage did not play a critical role in the stress distribution but had a profound effect on implant migration and rotation. Placement of the cage closer to the anterior side resulted in increased migration of the cage toward the anterior side of the spine. Notably, displacement measurements included translation, rotation, and deformation. The changes to the shape of the displacement distribution curves (Figure 5F) of the cages suggested that not only was the cage migrating, but also rotating and deforming as well. These results suggested that when considering the position of the cage, placing the cage closer to the posterior side to avoid anterior extrusion may lead to more warping and migration.

The angle of the superior vertebra affected stress distribution of the cage but had little effect on the migration. With increased rotation toward the posterior side, particularly above 10° rotation, there was an increase in the percentage of nodes above 65 MPa. There was higher maximum stress and strain with more rotation of the superior vertebra. In contrast, the displacement of the implant did not change with vertebral rotation, except for the sharpest rotation toward the posterior side, where we saw a flattening of the displacement distribution curve at values between 2.5 and 3.0 mm.

While this model specifically analyzed the anatomical geometry of the porcine spine, this approach of utilizing FE modeling is readily exportable and can be modified to understand vertebral anatomy for a wide variety of animals. Indeed, one of the major challenges of this field lies in the fact that there is a large variation in vertebral anatomy, both between different animal models and the human spine.^51^ Thus, FE modeling provides an alternative to rapidly screen multiple vertebral anatomies and corresponding implants. In fact, FE modeling has already been used extensively to analyze the mechanical behavior of implants over various physiological conditions.^32^, ^38^, ^54^

The current model heavily depended on the inputs and assumptions made to simplify the in vivo environment, particularly the material properties of PLA and boundary conditions of the model. While the mechanical properties of PLA were collected from mechanical testing, there was some discrepancy with the literature.^46^ This discrepancy may be due to differences in mechanical properties of PLA filament and structure fabricated via fused deposition modeling (FDM) compared to bulk PLA. Similarly, there may be alterations to the material properties of PLA after several weeks of implantation in vivo, particularly due to degradation or softening of PLA that is not captured here. Notably, we used two slightly different loads, ~2100 N and ~ 2300 N to assess implant migration and motion segment rotation. These parameters were chosen to optimize model convergence for these studies. While these nominal loads were slightly different, the trends for the effect of motion segment angle and implant placement were likely very similar between these two loading models. This model was also limited to instantaneous deformation and cannot capture results from cyclic loading or fatigue. A potential avenue for additional research is analyzing different materials and geometries for the cage, such as elastic or compliant materials as stiffer base materials tend to be more brittle, which may lead to failure.^21^ Furthermore, the implant used here had flat surfaces. In addition to selecting higher yield strength of our base material, we can modify the geometry of the cage to increase the contact area to minimize the stress concentration which will be important for future in vivo studies.

This model acts as a tool that can be used to quickly iterate cage designs prior to moving forward with in vivo or ex vivo testing. Engineers and scientists can utilize this simulation to predict the behavior of the implant and can customize the set‐up to run a multitude of parameters. Furthermore, personalized, patient‐specific spinal implants are already being used in surgical practice and demonstrate early efficacy in preventing complications.^55^, ^56^ In this regard, adaptation of the presented model may help determine and design optimal implant morphology based on patient anatomy and surgical goals.

## CONFLICT OF INTEREST STATEMENT

Lawrence J. Bonassar, Ibrahim Hussain, and Roger Härtl are consultants for 3DBio Therapeutics Corp., Lawrence J. Bonassar is co‐founder of 3DBio Therapeutics Corp. Remaining authors declare no conflict of interests. This work was partially funded by the Daedulus Fund.

## Supporting information


**DATA S1.** Supporting Information.

**VIDEO S1:** Video of FE model simulation with 2 mm displacement of IVD cage only. Vertebrae removed from view to better visualize the cage. *Source*: Video courtesy of Ansys, Inc. Notice the color bar scale is different from the main figures. In this video, the highlighted red represents Von Mises stress over 80 MPa.

**VIDEO S2:** Video of posterior view of FE model simulation with 2 mm displacement of IVD cage only. Vertebrae removed from view to better visualize the cage. *Source*: Video courtesy of Ansys, Inc. Notice the color bar scale is different from the main figures. In this video, the highlighted red represents Von Mises stress over 80 MPa.

**VIDEO S3:** Video of FE model simulation with 2 mm displacement of IVD cage with vertebrae. *Source*: Video courtesy of Ansys, Inc. Notice the color bar scale is different from the main figures. In this video, the highlighted red represents Von Mises stress over 80 MPa.
