# Supplementary material for: Finite element modeling to predict the influence of anatomic variation and implant placement on performance of biological intervertebral disc implants
Source: JOR Spine. 2023 Dec 27;6(4):e1307. doi: 10.1002/jsp2.1307 (PMC10751973; doi:10.1002/jsp2.1307)
Supplement: Supplementary file 1 — DATA S1. Supporting Information. [file JSP2-6-e1307-s001.pptx]

## Slide 1
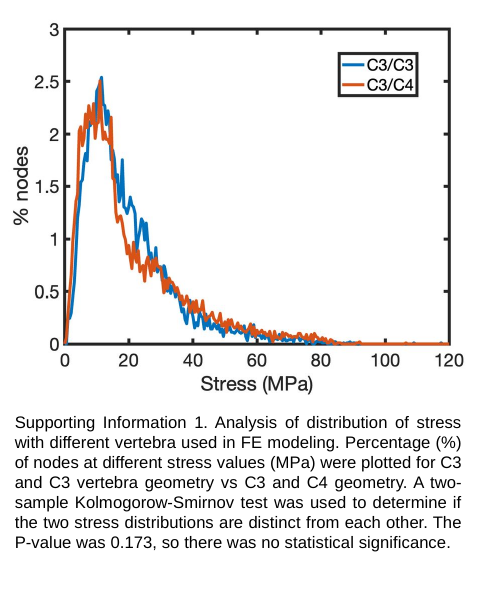

Supporting Information 1. Analysis of distribution of stress with different vertebra used in FE modeling. Percentage (%) of nodes at different stress values (MPa) were plotted for C3 and C3 vertebra geometry vs C3 and C4 geometry. A two-sample Kolmogorow-Smirnov test was used to determine if the two stress distributions are distinct from each other. The P-value was 0.173, so there was no statistical significance.

## Slide 2
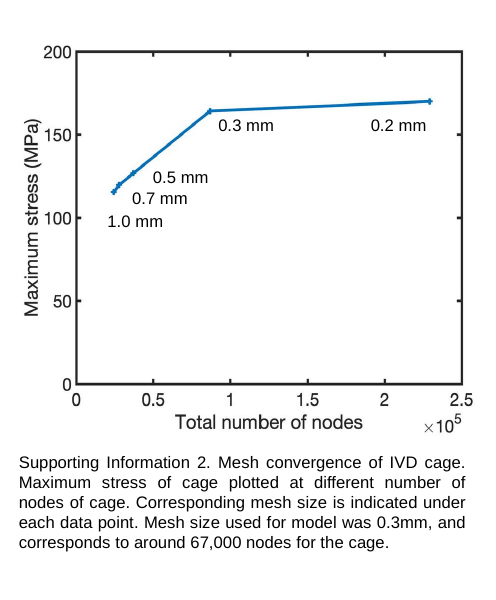

0.3 mm
0.2 mm
0.5 mm
0.7 mm
1.0 mm
Supporting Information 2. Mesh convergence of IVD cage. Maximum stress of cage plotted at different number of nodes of cage. Corresponding mesh size is indicated under each data point. Mesh size used for model was 0.3mm, and corresponds to around 67,000 nodes for the cage.

## Slide 3
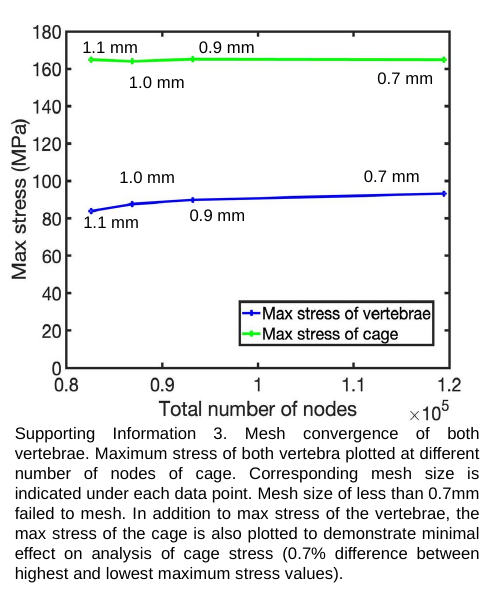

1.1 mm
0.9 mm
0.7 mm
1.0 mm
0.7 mm
1.0 mm
0.9 mm
1.1 mm
Supporting Information 3. Mesh convergence of both vertebrae. Maximum stress of both vertebra plotted at different number of nodes of cage. Corresponding mesh size is indicated under each data point. Mesh size of less than 0.7mm failed to mesh. In addition to max stress of the vertebrae, the max stress of the cage is also plotted to demonstrate minimal effect on analysis of cage stress (0.7% difference between highest and lowest maximum stress values).

## Slide 4
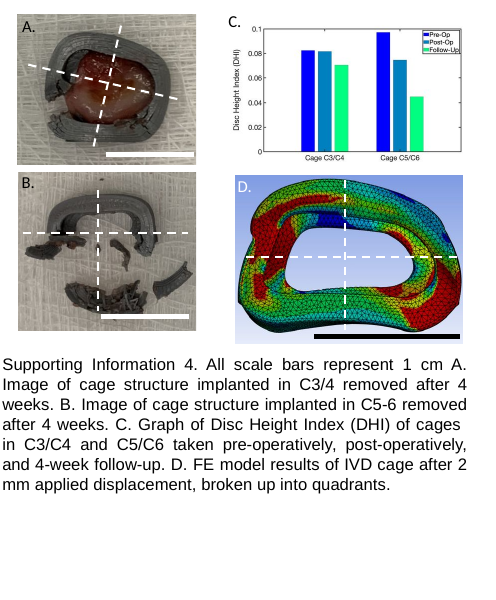

C.
A.
B.
B.
D.
C5-6
Supporting Information 4. All scale bars represent 1 cm A. Image of cage structure implanted in C3/4 removed after 4 weeks. B. Image of cage structure implanted in C5-6 removed after 4 weeks. C. Graph of Disc Height Index (DHI) of cages in C3/C4 and C5/C6 taken pre-operatively, post-operatively, and 4-week follow-up. D. FE model results of IVD cage after 2 mm applied displacement, broken up into quadrants.

## Slide 5
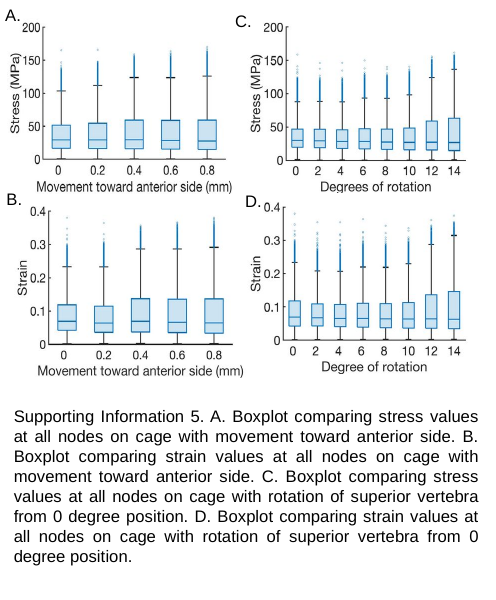

A.
C.
B.
D.
Supporting Information 5. A. Boxplot comparing stress values at all nodes on cage with movement toward anterior side. B. Boxplot comparing strain values at all nodes on cage with movement toward anterior side. C. Boxplot comparing stress values at all nodes on cage with rotation of superior vertebra from 0 degree position. D. Boxplot comparing strain values at all nodes on cage with rotation of superior vertebra from 0 degree position.
